# Income-related inequality in completed suicide across the provinces of Iran

**DOI:** 10.4178/epih.e2018012

**Published:** 2018-04-02

**Authors:** Mohammad Hassan Kazemi-Galougahi, Asieh Mansouri, Samaneh Akbarpour, Mahmood Bakhtiyari, Majid Sartipi, Rahmatollah Moradzadeh

**Affiliations:** 1 Department of Epidemiology and Biostatistics, School of Public Health, Tehran University of Medical Sciences, Tehran, Iran; 2 Hypertension Research Center, Cardiovascular Research Institute, Isfahan University of Medical Sciences, Isfahan, Iran; 3 Noncommunicable Diseases Research Center, Alborz University of Medical Sciences, Karaj, Iran; 4 Department of Epidemiology and Biostatistics, School of Health, Zahedan University of Medical Sciences, Zahedan, Iran; 5 Department of Epidemiology, School of Health, Arak University of Medical Sciences, Arak, Iran

**Keywords:** Socioeconomic factors, Suicide, Risk factors, Income, Iran

## Abstract

**OBJECTIVES:**

The aim of this study was to measure income-related inequality in completed suicide across the provinces of Iran.

**METHODS:**

This ecological study was performed using data from the Urban and Rural Household Income and Expenditure Survey-2010 conducted by the Iranian Center of Statistics, along with data on completed suicide from the Iranian Legal Medicine Organization in 2012. We calculated the Gini coefficient of per capita income and the completed suicide rate, as well as the concentration index for per capita income inequality in completed suicide, across the provinces of Iran.

**RESULTS:**

The Gini coefficients of per capita income and the completed suicide rate in the provinces of Iran were 0.10 (95% confidence interval [CI], 0.06 to 0.13) and 0.34 (95% CI, 0.21 to 0.46), respectively. We found a trivial decreasing trend in the completed suicide incidence rate according to income quintile. The poorest-to-richest ratio in the completed suicide rate was 2.01 (95% CI, 1.26 to 3.22). The concentration index of completed suicide in the provinces of Iran was -0.12 (95% CI, -0.30 to 0.06).

**CONCLUSIONS:**

This study found that lower income might be considered as a risk factor for completed suicide. Nonetheless, further individual studies incorporating multivariable analysis and repeated cross-sectional data would allow a more fine-grained analysis of this phenomenon.

## INTRODUCTION

Suicide is defined as a conscious effort to carry out self-damage that can lead to death [1]. Suicide is regarded as a process entailing suicidal ideation, planning, attempting, and—in catastrophic cases—completed suicide [2].

The annual global mortality rate of suicide is estimated to be 14.5 deaths per 100,000 population, which is equal to 1 million deaths every year [3]. Suicide is considered to be the third leading cause of mortality among people aged 15-44 [4] and the second leading cause of mortality in the 15-29 age group [5]. In 1998, suicide accounted for more than 2.0% of the global burden of disease and this proportion is predicted to increase to 2.4% by 2020 [2].

The median suicide rate for all countries of the world and the eastern Mediterranean region countries are 6.55 and 4.90 (per 100,000 population), respectively [6]. In 2009, the total numbers of suicide attempts and completed suicides in the Iranian population were 41,109 and 1,338, respectively. The rate of completed suicides (per 100,000) in Iran was 6.60 for males and 2.70 for females in 2010 [7]. According to a systematic review of studies on suicide, the prevalence of suicide in Iran is 9.40 (per 100,000), which is higher than in other eastern Mediterranean region countries [8]. The risk of suicide has been associated with many factors, such as mental disorders, unemployment, low income, ethnicity, genetic loading, sex, age, and life events [9,10]. These factors may influence attempted and completed suicide differently [3]. For example, in Ilam Province in Iran, which has a high prevalence of suicide, the attempted suicide rates were found to be highest in females, adolescents and young adults (15-24 years) and single individuals, while completed suicide was found to be most frequent in males, the elderly (≥ 65 years), divorced people, and widowers. Moreover, illiterate individuals had the lowest frequency of attempted suicide and the highest frequency of completed suicide [5]. In Iran, due to its nature as an Islamic country, religion can be considered as an important protective factor against suicide. Akbari et al. [2] reported that people with higher intrinsic religious beliefs committed suicide significantly less often. They explained this finding in terms of the greater social support received by such people in Iran [2].

There is considerable regional variation in the suicide rate. According to a systematic review, among studies that used the country as the areal unit for analysis, just 60% showed a significant association between suicide and socioeconomic status (SES). This percentage was 38, 40, and 43% for studies using areal units of the neighborhood, county, and state, respectively [11]. Another study showed that the mortality rate due to suicide varied across dongs (the smallest administrative unit) in Seoul. In that study, the lowest rate of deaths by suicide was seen in the most affluent dongs [12]. There is a similar pattern in Iran, with the highest suicide rate in Ilam, Kermanshah, and Lorestan Provinces [7,13].

The methods that people choose for suicide vary across geographical region [5]. For example, self-burning is rare in developed countries, while it is a common method in Iran. According to Mirhashemi et al. [14], the widespread domestic storage of flammable fluid tanks for cooking and heating can explain this divergence in methods of suicide across countries. Shirazi et al. [15], identified drugs, self-burning, and poisoning as the 3 most common methods of suicide in a systematic review and meta-analysis of suicide in Iran.

Based on previous studies of suicide in Iran, it can be concluded that suicide does not show an equal demographic and sociocultural distribution in Iran. The concept of inequality allows these differences to be elaborated in a subtler fashion. Simply put, when a health problem does not show an equal distribution according to socioeconomic factors (e.g., sex, age, education, income, etc.), it exhibits inequality [16]. Economic constraints have frequently been reported to be among the most important contributors to suicide in the literature on Iran. According to a previous study, 12% of suicides in Iran are related to this factor [14]. Therefore, we investigated the relationship between economic status and suicide in a more specific manner by estimating income-related inequality indices in this study. This technique can be a useful tool to gauge and improve suicide reduction interventions. These inequalities were investigated using provincial data in this study. In Iran, the province is the largest administrative unit within the country. The units of administrative divisions in Iran include (from large to small) province, county, sector, rural district, and city. The number of each of those divisions was 31, 429, 1,057, 2,589, and 1,245, respectively, according to the most recent report of the Statistical Center of Iran [17].

## MATERIALS AND METHODS

This was an ecological study with the province, an aggregated unit, as the unit of analysis. We linked 2 datasets that were separately collected. The suicide data were obtained from a survey by Shojaei et al. [7] intended to determine the number of suicide deaths registered with the Iranian Legal Medicine Organization. In this survey, 3,513 suicide deaths in 2010 were analyzed. Based on the Iranian Legal Medicine Organization database, suspected suicide cases whose corpse was referred to a legal medicine center were investigated. Cases in which family members or companions confirmed that suicide had been committed and cases that seemed to be suicide based on the early examination were included in the research. Cases where suicide was not confirmed after necropsy and a physician’s investigation were excluded. The relevant questions were asked of the family or companions of the decedent in the intake section of the legal medicine centers. The completed questionnaires were sent to the provincial capital for data entry and the collected information was sent to a central office. If incomplete answers were given to some of the questions that were asked, the files were re-investigated and in some cases, family members were contacted to obtain more complete information [7]. As a socioeconomic indicator for estimating inequalities, per capita income in the various provinces of Iran in 2010 was obtained from the Households Income and Expenditure Survey conducted in 2010 by the Statistical Center of Iran [18]. This survey was carried out on a sample of 18,701 households in urban areas and 19,584 households in rural areas, and targeted all private and collectively settled households in the included areas. A 3-stage cluster sampling method with strata was used in the survey. In the first stage, the census areas were classified and selected. The selection of urban and rural blocks and sample households corresponded to the second and third stages, respectively. The sample size was optimized to estimate the average annual income and expenditures of the sample households. In order to obtain more representative estimations of the whole year, the samples were evenly distributed across the months of the year [18]. Since the results of this survey were published according to urban and rural household income, we considered the proportion of urbanity and the mean household size in each province to calculate the per capita income of all provinces.

### Statistical analysis

Stata version 12/SE (StataCorp., College Station, TX, USA) was used for the statistical analysis. The mean (standard deviation [SD]) of the suicide rate was calculated by summing the rates of suicide in each province and dividing it by the population of that province. We divided the per capita income among provinces into 5 quintiles and estimated the mean (SD) suicide rate in each quintile. As a non-specific inequality index, we calculated per-quintile suicide rate ratios considering the fifth (richest) quintile as the reference group.

Inequality was also estimated using the Gini index and the concentration index and displayed with a Lorenz curve and concentration curve, respectively.

### Gini index

The Gini index is a univariate index of inequality that takes on values between 0 (perfect equality) and 1 (perfect inequality). Twice the value of this index is interpreted as the expected mean difference between 2 randomly selected persons in the population [19].

### Lorenz curve

The Gini index can be derived from a Lorenz curve. In a Lorenz curve, the cumulative percentage of the outcome variable (on the vertical axis) is plotted against the cumulative percentage of the population (on the horizontal axis). The Gini index can be calculated as the area between the diagonal (45°) line and the Lorenz curve [20].

### Concentration index

The concentration index is a bivariate index of inequality, calculated as twice the covariance of the health-related outcome variable and the fractional rank in the SES-related variable divided by the mean of the health-related outcome [21]. This index varies between -1 and 1, with positive values implying that the variable is concentrated in the affluent subpopulation and negative values implying the opposite. A concentration index of 0 implies that the variable has an equal distribution [22].

### Concentration curve

The concentration curve plots the cumulative percentage of the outcome variable (on the vertical axis) against the cumulative percentage of the population, ranked by the SES-related variable, beginning with the most disadvantaged person (on the horizontal axis) [22].

While the Lorenz curve is always placed somewhere below the diagonal [20], the concentration curve can lie above the diagonal when the outcome is typically more common or larger in magnitude among the worse-off or below the diagonal when the outcome is typically more common or larger in magnitude among the better-off [23]. If either curve coincides with the diagonal, perfect equality is considered to be present (Gini index= 0, concentration index= 0).

## RESULTS

**Figure 1.**
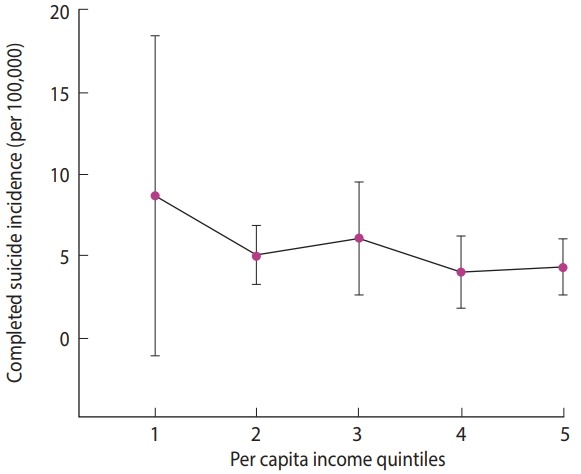
Estimated completed suicide rate (per 100,000) with 95% confidence intervals in per capita income quintiles for the provinces of Iran, 2010.

The mean± SD completed suicide rate was 5.65± 3.61. The ranges of per capita income in the poorest quintile (quintile 1) to the richest quintile (quintile 5) were 17,555,200-25,797,871, 25,797,872-27,168,924, 27,168,925-30,032,150, 30,032,151-32,358,134 and 32,358,135–48,583,808 rials, respectively (USD 1 in 2010 ≈ 10,500 Iranian rials). The mean completed suicide rate by per capita income quintiles is presented in Table 1. We estimated the rate ratios and respective 95% confidence intervals (CIs) for different quintiles, with the richest quintile used as the reference category. According to Table 1, the mean suicide rate was the highest in the first quintile (the provinces with the lowest per capita income) with a slight descending trend from the first quintile to the fifth quintile (the provinces with the highest per capita income). This trend is illustrated in Table 1 and Figure 1.

**Figure 2.**
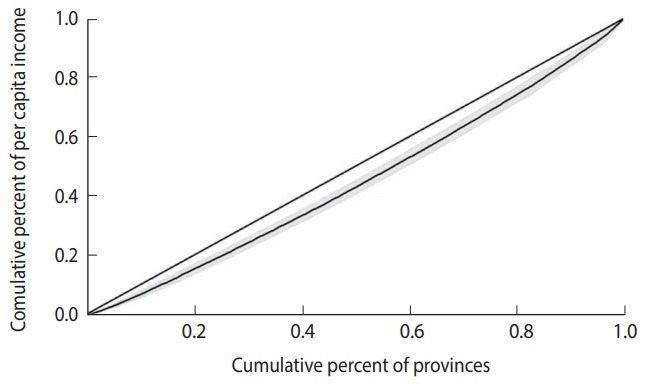
Lorenz curve for per capita income in the provinces of Iran, 2010.

**Figure 3.**
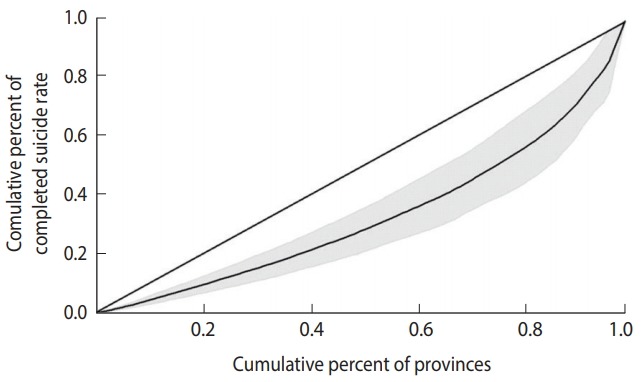
Lorenz curve for the completed suicide rate in the provinces of Iran, 2010.

The Gini index for per capita income and the completed suicide rate in the provinces of Iran was 0.10 (95% CI, 0.06 to 0.13) and 0.34 (95% CI, 0.21 to 0.46), respectively. Figures 2 and 3 show the Lorenz curves for per capita income and the completed suicide rate in the provinces of Iran in 2010. These indices and figures imply that both per capita income and completed suicide rates showed statistically significant inequality in Iran.

**Figure 4.**
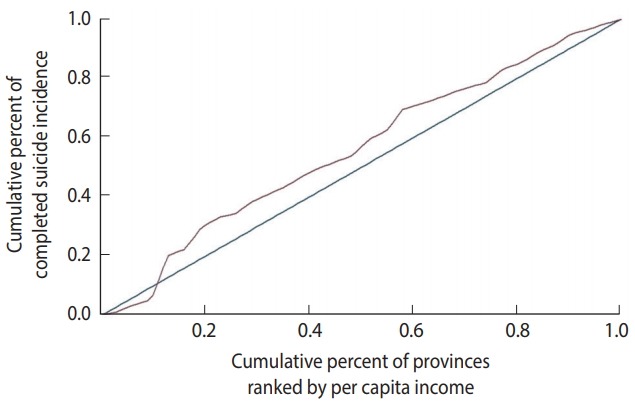
The concentration curve of completed suicide in the provinces of Iran, 2010.

**Figure 5.**
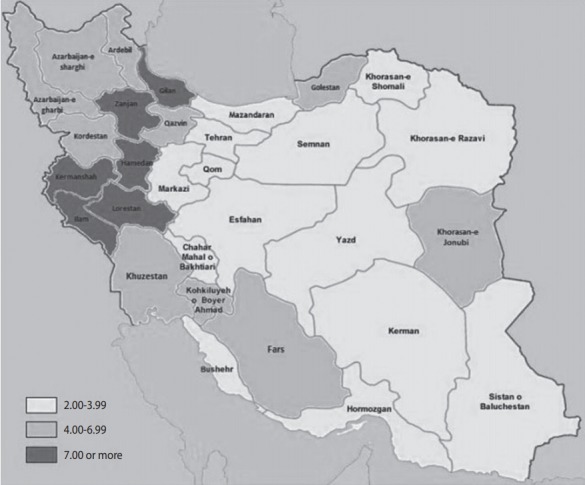
Completed suicide rate (per 100,000) in the provinces of Iran, 2010.

The concentration index of suicide was -0.12 (95% CI, -0.30 to 0.06). Figure 4 shows the concentration curve of completed suicide for the provinces of Iran, indicating a non-significant slight concentration of completed suicide among the provinces with lower per capita income. Figure 5 illustrates the distribution of completed suicide in Iran (incidence rate per 100,000) by provinces in 2010. As shown, completed suicide did not have an equal distribution in the provinces of Iran.

## DISCUSSION

To the best of our knowledge, this was the first study to investigate economic inequalities in completed suicide at the provincial level in Iran and to address the information gap on the distribution of suicide by economic status. According to the last census in Iran, the population of Iran is more than 79 million. Additionally, the 30- to 64-year-old age group accounts for the largest proportion of the population, and the most common family size is 3. The majority of Iranians 10 years or older are married (females, 64.3%; males, 63.3%), and the literacy rate is 94.7% in Iran.

We found slight inequality in per capita income and severe inequality in the completed suicide rate among the provinces of Iran in terms of Gini coefficients.

This study exhaustively investigated the inequality in completed suicide. We found a roughly constant reverse association between the completed suicide rate and per capita income in the provinces. The estimated rate ratios of completed suicide for different per capita income quintiles (with the richest quintile as the reference category) implied that only the poorest quintile (i.e., the first quintile) had a statistically significant rate ratio (p= 0.004). There were no significant rate ratios in other quintiles.

The concentration index of completed suicide was negative and statistically non-significant. However, Gini coefficient, as other inequality index, indicated completed suicide has a vigorous disparity in Iran. Therefore, completed suicide was found to be concentrated in provinces with lower per capita income. This finding is consistent with results obtained from other ecological studies around the world, which have mostly showed an inverse association between suicide and income [24-26]. Puzo et al. [27] observed that suicide risk significantly and progressively increased with lower levels of income. According to a systematic review, weak economic status, low wealth, and unemployment are associated with suicidal behaviors [28]. A study in Japan found that fluctuations in suicide rates could partially be explained by income inequalities. That study concluded that it is important to consider income distribution and employment status when setting up suicide reduction programs [29]. In another study in 15 European countries, the suicide rate was negatively related to geoeconomic categorization, with rich areas having the lowest rates and poor areas the highest [30].

Although the mechanisms of this relationship are unknown, income inequality can affect individuals by strengthening depression and feelings of hopelessness. Social and economic deprivations in poorer people may make them disappointed and bring them closer to deciding to commit suicide. Poverty in the developing world may increase the suicide rate through factors such as financial problems, stress, mental health problems, lower SES, and lower access to health care services [31]; thus, these factors should be investigated in more detail.

When we plotted a map of the geographic distribution of completed suicide, we observed a higher incidence of completed suicide in the western provinces of Iran than in the central and eastern provinces. This finding is expectable, since these provinces are socioeconomically more deprived than the central or eastern provinces. In a study in Taiwan, suicide was similarly more common in suburban regions than in the central cities [32]. There is considerable geographical variation in the suicide rate in the US with a greater frequency of migration, divorce, and unemployment have been found to have higher rates of suicide [33].

Although we used only per capita income as an indicator of SES in this study due to an inability to access other indicators, our results are in accordance with those of multiple previous studies. In a systematic review of the relationship between SES and suicide, 61 of the 221 studied analyses used income as a measure of SES. Fifty percent of these studies showed a significant reverse relationship between income and suicide [11].

### Strengths and weaknesses

Our study has some strengths. First, to our knowledge, this is the first national provincial-level study to investigate income inequality and suicide in Iran. Second, the present study was conducted with accurate and valid data on suicide and income based on the Iranian Legal Medicine Organization and the Statistical Center of Iran. However, similarly to other studies, the present study has some weaknesses. The results of this study are restricted by the scope of our data. Suicide mortality data do not reflect incomplete suicide attempts. The association of income with incomplete suicides may be quite different from the relationship with completed suicide. Additionally, we did not have access to other socioeconomic indicators on the level of the provinces of Iran. Income may not be a comprehensive indicator of SES. In addition, the lack of access to data on other determinants on the provincial level meant that we could not perform an adjusted analysis. Therefore, we suggest conducting an adjusted analysis of inequality in the future with more indicators of SES. The final major limitation of this study is the use of aggregated data, with the concomitant possibility of ecological bias.

In conclusion, the present study suggests that income inequality likely increases the risk of completed suicide in Iran. Therefore, reducing income inequality, followed by improving provinciallevel SES, is advisable. Although this study suggests that lower income may be a risk factor for this phenomenon, more studies with multivariate analyses and repeated cross-sectional data to allow monitoring over time can help researchers to explore this issue in more detail.

## Figures and Tables

**Table 1.** Estimated completed suicide rate (per 100,000), its RR and 95% Cl in per capita income quintiles for the provinces of Iran, 2010

Quintiles	Completed suicide rate (per 100,000, mean±SD)	RR (95% CI)	p-value
1	8.73±9.31	2.01 (1.26, 3.22)	0.004
2	5.05±1.70	1.16 (0.69, 1.97)	0.66
3	6.13±3.79	1.41 (0.86, 2.37)	0.22
4	4.03±2.03	0.93 (0.53, 1.62)	0.91
5	4.33±1.60	1.00 (reference)	

RR, rate ratio; CI, confidence interval; SD, standard deviation.
